# Adelmidrol to fight upper airways inflammation in children: a pilot case control study to safety and efficacy

**DOI:** 10.1007/s00405-025-09375-z

**Published:** 2025-04-21

**Authors:** Antonio Della Volpe, Chiara Bruno, Pietro De Luca, Massimo Ralli, Arianna Di Stadio

**Affiliations:** 1 Otolaryngology Department, Santobono-Posillipon Hospital, Via Mario Fiore 6, 80129 Naples, Italy; 2 Department of Otolaryngology, Tiberina-Isola Gemelli Hospital, Via di Ponte Quattro capi, 39, 00186 Rome, Italy; 3 Organ of Sense Department, University La Sapienza of Rome, Via del Policlinico 155, 00161 Rome, Italy; 4 ENT Department, University of Campania “Luigi Vanvitelli”, Naples, Italy

**Keywords:** Ademidrol, Upper respiratory infection, Pediatrics, Adenoids, Tonsils, Tympanic membrane

## Abstract

**Purpose:**

Inflammations of the upper respiratory tract (URT) are common both in adults and children and they are generally treated using aerosol therapy with mucolytic medications and steroids. When these inflammations affect children, the treatment must be rapid and resolutive to prevent complications. Steroids present some contraindications, i.e. alteration of smell, that must be considered especially in children. Therefore, alternative treatments that have similar efficacy but limited adverse effects should be considered. This study aims at evaluating the efficacy of Adelmidrol to treat inflammation of the URT in children.

**Methods:**

Case-Control study. Control group used standard treatment for URT inflammation (mucolytics and steroids); treatment groups were treated by Adelmidrol spray. Sixty children (age range 2.5–4.5 years) were randomly assigned to (i) control group, (ii) treatment group 1 (TG1)- Adelmidrol nasal spray only and (iii) treatment group 2 (TG2), in which Adelmidrol was administered in both nasal and oral spray solution. The URT and the tympanic membrane were evaluated at T0, T1 (30 days) and T2 (90 days). The treatments were performed for 90 consecutive days.

**Results:**

At the end of the treatment, TG2 (combination of nasal and oral sprays) had the best outcomes both on URT findings (χ²: *p* = 0.0004) and tympanic membrane conditions (χ²: *p* = 0.03). TG1 showed similar outcome of CG.

**Conclusions:**

These preliminary results in our group of 60 children showed that Adelmidrol had the same efficacy of standard treatment when used as nasal spray only and was better than the standard treatment when used combining nasal and oral sprays. The molecule seems to offer the same benefit of standard treatment without side effects. If confirmed on a larger sample, the use of Adelmidrol could be suggested as an alternative to traditional treatment for the inflammation of URT in children.

## Introduction

Inflammations of the upper respiratory tract (URT) are a common condition in the general population. However, children suffer from this condition more than adults because of their developing immune system [1] and their specific anatomical characteristics, such as a shorter and flatter Eustachian Tube (ET) [2].

Different clinical trials [2, 3] have shown that by using natural immune stimulant compounds it is possible to reduce the incidence of infection in the URT and the time to complete recovery. However, these trials only evaluated the efficacy of systemic natural compounds, also called nutraceuticals [2, 3], and not topical treatments.

During the COVID-19 pandemic, the use of natural compounds including nasal spray has been proposed because it could improve the nasal environment [4] and reduce local inflammation. The combination of these two aspects could improve the local immune response confining Sars-CoV2 and avoiding viral spread in the lungs [5, 6].

Steroids, although largely used to fight nasal inflammation, have an immune-suppressant action that can increase the risk of lungs infections especially in long-time use [7].

Palmitoylethanolamide (PEA), a cannabinoid mimetic molecule that has the same anti-inflammatory effects of cannabinoid, but it is an amide - has been shown to effectively inhibit viral replication and spread in case of Sars-CoV2 infection, highlighting its potential role as a therapeutic agent to fight COVID-19 [8].

PEA has well-known anti-inflammatory and antioxidant proprieties [8, 9], in addition to this recently shown anti-viral effect [8]. Adelmidrol is a precursor of PEA, and we hypothized that it might hae similar proprieties of PEA. In fact, Adelmidrol has shown capacities of limiting the infiltration of inflammatory cells, inhibiting the activity of Myeloperoxidase and the excessive expression of pro-inflammatory cytokines (IL-6, IL-1β, TNF-α, and TGF-1β) [9]. These phenomena are present in any type of inflammation, including URT. We speculate that the anti-inflammatory capacities of Adelmidrol, a natural compound with no adverse effects, could have a role in fighting URT inflammation.

One of the more interesting characteristics of Adelmidrol is the modulation of mastocyte action without immune suppression [9]; nasal mastocytes have an important role both in acute and chronic nasal inflammation [10].

This pilot study aims to compare the outcome of patients with URT inflammation by using two different combinations of Adelmidrol (pharyngeal spray and nasal spray) versus traditional standard treatment that combines mucolytic and steroids specifically looking at the nasopharynx and tympanic membrane conditions. The pharmaceutical names of the products with Adelmidrol are Oridrol ^®^ for pharyngeal spray and Rinidrol ^®^ for nasal spray.

## Materials and methods

This pilot study was conducted in the Department of Otolaryngology of a tertiary pediatric referral center from January 2023 to December 2023. All procedures were approved by the local Institutional Review Board (SBP38 and 11-11-2022) and conducted in accordance with the ethical principles outlined in the Declaration of Helsinki. The parents of participating children signed a written informed consent to authorize the enrollment of their child in the study.

Sample size calculation was performed by using an on-line calculator (https://clincalc.com/stats/samplesize.aspx). The calculator determined that 15 patients were required to achieve a 5% margin of error at a 95% confidence level and have 80% power. Because the study needed 3 study groups, and dropout could be possible, 60 children were enrolled. Children enrolled (age range 2.5–4.5 years; 31 females and 29 males) were suffering from recurrent inflammation of the upper respiratory tract, in particular were affected by adeno-tonsillar hypertrophy and recurrent otitis media. We opted for 60 patients, the minimally statistically significant number, because this pilot study wanted to evaluate safety and the efficacy of the Adelmidrol before performing a blinded clinical trial.

The same otolaryngologist with 30 years of experience (ADV) evaluated all children at each follow-up (T0 baseline at the moment of enrollment, T1 one month after treatment, and T2 three months after treatment at the end of the study).

The TM was analyzed by using a Sensera microscope (Zeiss, Oberkochen, Germany). The state of the TM was photo-recorded and then scored from 1 to 3 as follows: 1 = hyperemic TM, 2 = opaque TM, and 3 = healthy TM (parameter 1 in Table 1). In case of asymmetric findings between the two ears, the worse one was considered.

**Table 1:** Summary of the clinical assessment used in the study

1	Microscopic evaluation and photo-recording of tympanic membrane (TM), using the following score: TM hyperemic = 1 TM opaque = 2 TM healthy = 3
2	*Nasopharingeal fibroendoscopy* (using the Cassano’s score to assess the adenoid tissue’s state) **Grade I**: fiberoptic endoscopy showed adenoid tissue occupying < 25% of choanal space. **Grade II**: adenoid confined to the upper half of nasopharynx, with sufficiently free of choana and visualization of tube ostium. **Grade III**: adenoid vegetation occupied about 75% of the nasopharynx with partial involvement of tube ostium and considerable obstruction of choana area. **Grade IV**: complete obstruction of rhynopharynx with total occlusion of tubaric ostium.

Subsequently, the physician investigated the state of the nose and pharynx using flexible fiberoptic endoscopy (Storz, Tuttlingen, Germany) and an Olympus CV-170 camera (Olympus, Shinjuku, Tokyo, Japan) to determine the presence and volume of adenoid tissue. The findings were classified using the Cassano assessment scale, which ranges from 1 to 4 [11] (parameter 2 in Table 1).

Because adenotonsillar hypertrophy is not the only sign of inflammation of the URT, we combined the Cassano score with a score for retronasal discharge. The presence (1) or absence (0) of retronasal discharge was noted. In cases of presence, a score of 1 indicated slight retronasal discharge, while a score of 2 indicated abundant discharge. This score was added to the Cassano score to obtain a single number.

The combination of the Cassano score and the score for retronasal discharge determined the outcome referred to as “upper respiratory tract inflammation.”

### Randomization

Randomization was performed at the baseline evaluation (T0) using a computer program available online (https://www.randomizer.org); the program assigned consecutive number at each subject included in the study. Following this numeration the children were assigned to the different groups as follow number 1, 4, 7 etc. to control group; 2, 5, 8 etc. to treatment group 1 and number 3, 6, 9 etc. to treatment group 2. This numeric assignment was performed until reaching 20 subjects for group.

### Group treatment details

*Control group (CG*) used the standard therapy (nasal wash with saline solution and nasal aerosol with Fluticasone and Mucolytic) suggested by international consensus (2). The following therapeutic scheme was used: nasal wash three times a day for 3 consecutive months combined to nasal aerosol for 10 days, then 20 days suspension. The aerosol treatment was performed with same therapeutical scheme each month for. 3 months in total. *Treatment group 1 (TG1)* (20 subjects) was treated with Rinidrol^®^ nasal spray (isotonic saline solution containing 2% Adelmidrol and 0.2% Hyaluronic Acid), using 2 sprays in each nostril 3 times a day until symptoms resolved for 90 consecutive days (3 months).

Patients in *treatment group 2* (TG2) (20 subjects) were treated with a combination of Rinidrol^®^ (2 sprays in each nostril 3 times a day) and Oridrol^®^ oral spray (aqueous solution containing 2% Adelmidrol and 10% Echinacea Angustifolia extract), using 2 sprays on the oral cavity mucosa 3 times a day for 3 consecutive months.

The posology of Rinidrol (https://www.epitech.it/product/6035/it) and Oridrol (https://www.epitech.it/product/6037/it) was the one suggested by the producer (Epitech SPA) for the treatment of nasal and/or pharyngeal inflammation.

To improve the quality of treatment, the otolaryngologist explained to the parents the correct modalities to use the spray (position of the spray parallel to the nostril and parallel to the tongue). Moreover, to monitor the correct adherence to the treatment or manage adverse event related to the use of the treatment a telephone check was made weekly, and a dedicated otolaryngology nurse was available 24 h by phone for questions and support.

The following inclusion and exclusion criteria were used:

*Inclusion criteria* were age between 3 and 6 years, normal immunological state, and absence of neurological, psychiatric, or psychological disorders.

*Exclusion criteria* were previous ear surgery, previous nasopharynx or pharynx surgery, patients affected by metabolic syndromes (e.g., diabetes), and those children whose parents did not agree to their participation in the study. Patients who needed antibiotics during the observation period were also excluded.

Demographic data including gender, age, pre-existing conditions, previous surgery in other districts were collected. In case of adverse event using Adelmidrol, the event was noted and the treatment immediately suspended.

### Explanation of evaluation method for URT and TM findings

Two numeric methods were used to evaluate our patients both with TM and the adenoid hypertrophy (Cassano Score). The latter was modified by adding another value for retronasal discharge (see before) by creation of a modified Cassano score. The improvement was considered the reaching of high score in case of TM, where 3 correspond to normality, while in the context of the modified Cassano assessment the amelioration was based on the decrease of the numeric value (1 = normal).

### Statistical analysis

Statistical analysis was performed by one of the authors (A.D.S.), certified in biostatistics. One-way ANOVA was performed to analyze the score variation ***within*** the groups (TG1, TG2, and CG) at three observational points (T0, T1, and T2) for the otoscopy findings. The same test was repeated to evaluate the variance of fibro-endoscopy findings at the same observation times of the otoscopy. A Holm–Bonferroni (HB) ad hoc test was performed for each one-way ANOVA. Within tests were performed to evaluate the changes of the observed parameters in the same group, to understand if the treatment used was beneficial. Chi-Square (χ) was used to compare the data ***between*** groups. The statistical analysis was performed by STATA^®^. p value was considered statistically significant < 0.05.

## Results

### General

Fifty-four (54) patients completed the study. Twenty of them were in CG (0% drop off) (13 females and 7 males, average age 4.3 *±* 1.2 years), 14 in TG1 (30% drop off) (8 females and 6 males, average age 3.6 *±* 1.1 years) and 20 in TG2 (0% drop off) (10 females and 10 males, average age 3.8 *±* 1 years).

Six patients (2 females and 4 males) in TG1 were excluded from the final analyses because they needed antibiotics during the observation time.

None of the patients in the treatments group presented adverse reactions to the Adelmidrol nor to Oridrol ^®^ or Rinidrol ^®^ (Table 2).

**Table 2:** Demographic characteristics of the observed population

Group	CG	TG1	TG2
Patients (n)	20	14	20
Gender (n)	13 females 7 males	8 females 6 males	10 females 10 males
Age (years, mean ± SD)	4.3 ± 1.2	3.6 ± 1.1	3.8 ± 1
Upper airways inflammations (mean ± SD)			
T0 T1 T2	1.6 ± 0.5 0.8 ± 0.6 0.8 ± 0.8	1.5 ± 0.8 1.1 ± 0.7 1 ± 0.6	1.7 ± 0.8 0.6 ± 0.8 0.3 ± 0.6
Ear findings (mean ± SD)			
T0 T1 T2	1 ± 0 1.1 ± 0.4 1.3 ± 0.5	1.6 ± 0.5 1.8 ± 0.7 2.5 ± 0.6	1.2 ± 0.6 1.8 ± 0.3 2.8 ± 0.5

CG, control group; TG1, therapeutic group 1; TG2, therapeutic group 2; SD, standard deviation

### Within group

#### Inflammation of the upper respiratory tract

***CG*** patients treated by standard therapy improved their URT inflammation score with statistically significant value (ANOVA: *p* = 0.002) comparing T0 (1.6 *±* 0.5) and T1 (0.8 *±* 0.6) (BH: *p* < 0.05) and T0 and T2 (0.8 *±* 0.8) (**BH**: ***p*** < **0.05**); no statistically significant differences were observed between T1 and T2 (BH: *p* > 0.05). This result means that the use of standard therapy allowed to the patients to reduce adenotonsillar inflammation including a reduction of retronasal discharge. The obtained benefit was already visible at T1; however, despite an ulterior improvement at T2 this was not such strong enough to determine statistically significant changes.

***TG1*** Patients treated with Rinidrol (Adelmidrol nasal spray) showed a slight improvement of the URT inflammation comparing T0 (average 1.5 *±* 0.8), T1 (average 1.1 *±* 0.7) and T2 (average 1 *±* 0.6), despite not statistically significant. Despite the improvement, the reduction of adenotonsillar hypertrophy and the retronasal discharge was minimal both at T1 and at the end of the therapy (T2).

**TG2**: Patients treated with the combination of Rinidrol and Oridrol (Adelmidrol oral spray) had a reduction of URT inflammation (reduction of adenoid hypertrophy and retro nasal secretion) with a statistically significant improvement (ANOVA: *p* < 0.0001). The improvement was observed comparing T0 (1.7 *±* 0.8) and T1 (0.6 *±* 0.8) (BH: *p* < 0.01) and T0 and T2 (0.3 *±* 06) (BH: *p* < 0.01); no statistically significant differences were observed between T1 and T2 (BH: *p* > 0.05). The combination of the two sprays was able to substantially reduce the inflammation in the adenoid and tonsil tissues with total disappearing of retronasal discharge. The improvement was already important at T1, and although it continued to improve at T2 this amelioration was not statistically important.

#### Tympanic membrane findings

CG: No statistically significant improvement of TM findings was observed comparing T0 (1 *±* 0) and T1 (1.1 *±* 0.4) and T0 and T2 (1.3 *±* 0.5). The condition of the TM did not change by using standard treatment.

TG1: A statistically significant improvement of TM findings (ANOVA: *p* < 0.0001) was found comparing T0 (1.6 *±* 0.5) and T1 (1.8 *±* 0.7) (HB: *p* < 0.05) and T0 and T2 (2.5 *±* 0.6) (HB: *p* < 0.01). Statistically significant differences were observed comparing T1 and T2 (HB: *p* < 0.01). The use of Adelmidrol spray was able to improve the condition of TM after 1 month of treatment in most of the subjects; this improvement was extended to the other children after three months (T2) with return to normal TM appearance.

**Fig. 1:**
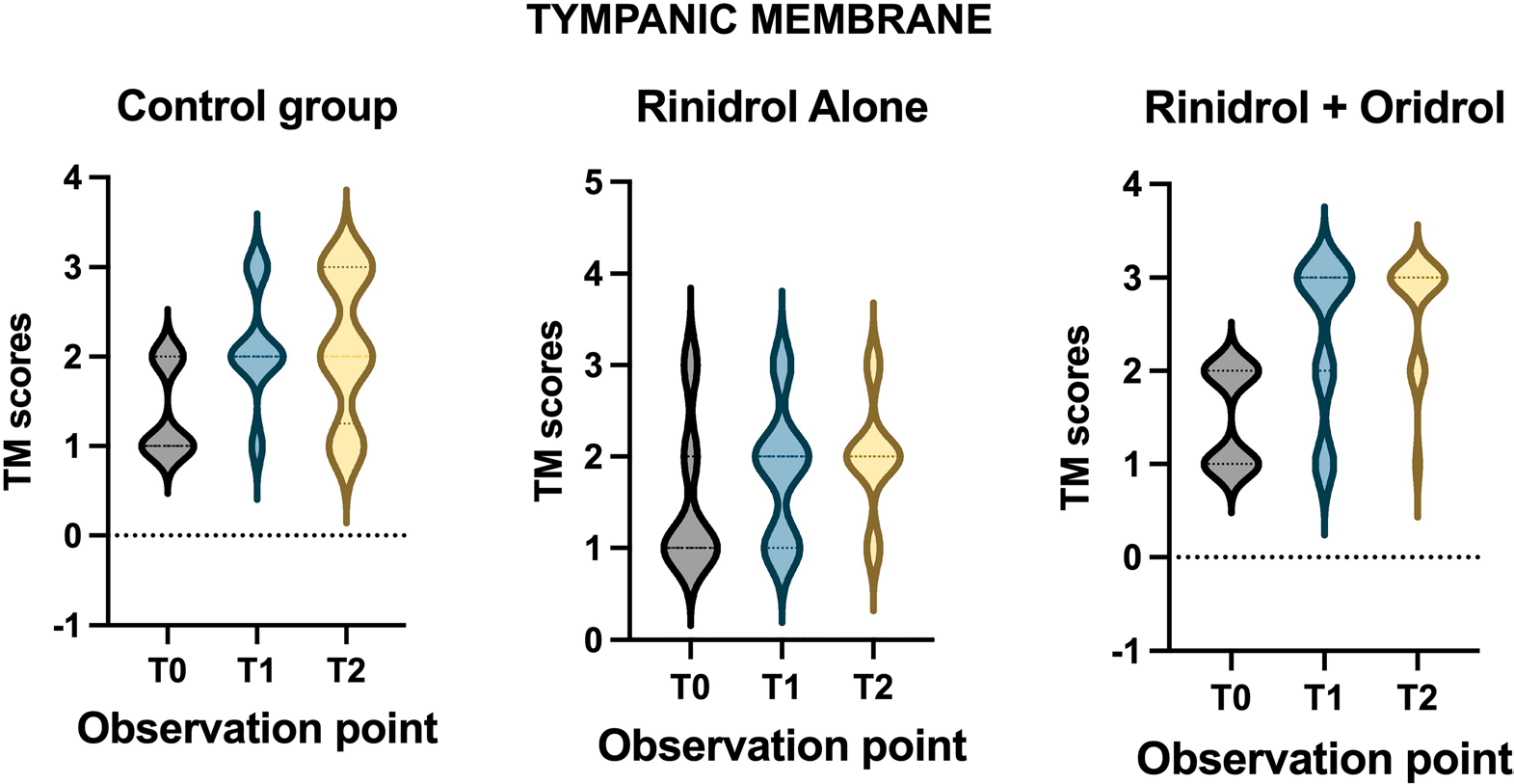
Within group comparison of tympanic membrane findings in control, TG1 (Rinidrol) and TG2 (Rinidrol + Oridrol)

TG2: Statistically significant improvement of TM findings (ANOVA: *p* < 0.0001) was observed comparing T0 (1.2 *±* 0.6) and T1 (1.8 *±* 0.3) (HB: *p* < 0.01) and T0 and T2 (2.8 *±* 0.5) (HB: *p* < 0.01). No statistically significant differences were observed between T1 and T2 (HB: *p* > 0.05) (Fig. 1). The combination of sprays substantially improved the condition of TM after 1 month of treatment; the improvement was such notable that despite further improvement at the end of the treatment, no statistically significant changes were identified.

### Between groups

Statistically significant differences, in term of improvement, were observed between CG, TG1 and TG2 (χ: *p* = 0.0004) comparing the upper airways findings at T1 and T2 with T0. These data mean that although all patients obtained the improvement of their adenotonsillar hypertrophy, these improvements were different among the three observed groups.

**Fig. 2:**
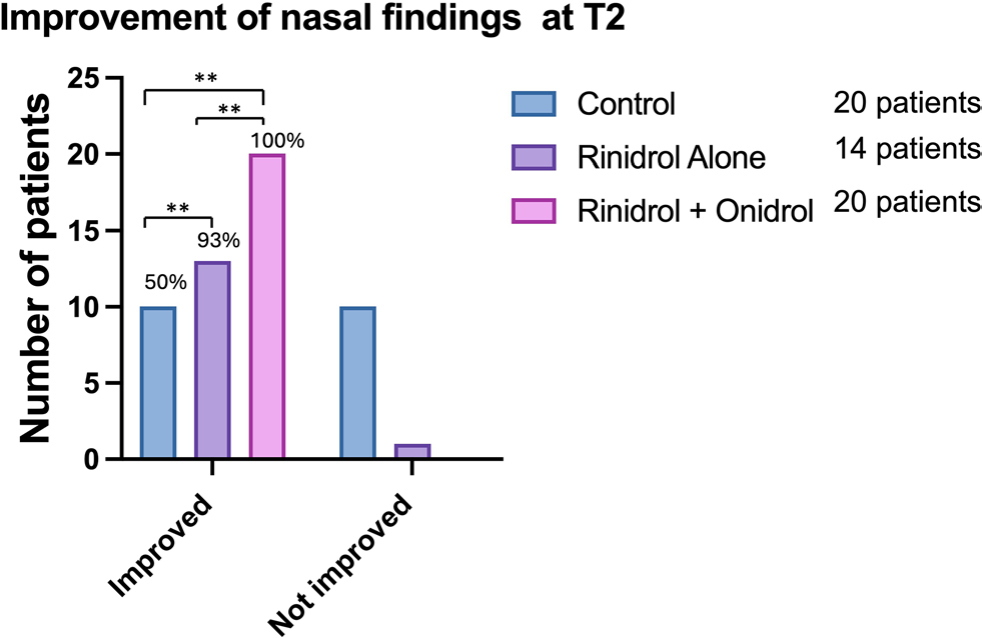
Between group comparison of nasal findings. The number over the columns represent the percentage of improvement. “**” *p* < 0.01

In details, 2 patients in the CG, 6 in TG1 and 12 TG2 improved their nasal findings comparing T0 and T1; at T2 8 patients improved from T1 in the GC, 7 in TG1 and 8 in TG2. At the end of the three-month period (T2) 10 children (50%) improved nasal outcomes in CG, 13 (92.8%) in TG1 and 100% in TG2 (Fig. 2).

**Fig. 3:**
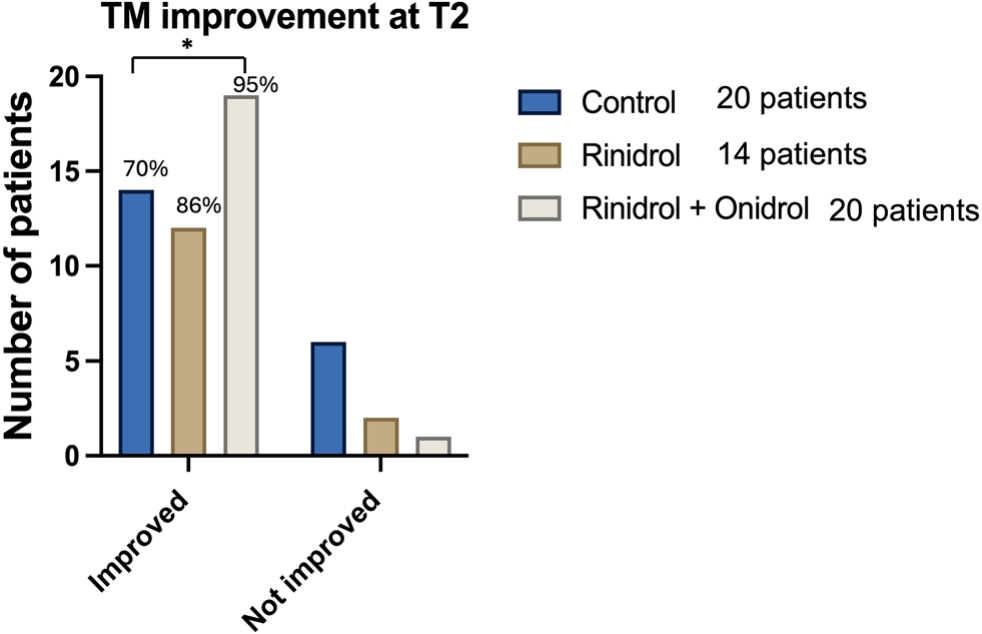
Between group comparison of tympanic membrane findings. The number over the columns represent the percentage of improvement. “*” *p* < 0.05

Statistically significant differences were observed between CG, TG1 and TG2 (χ: *p* = 0.03) comparing the TM findings at T1 and T2 in term of improvement from T0. Specifically, at T1 6 (30%) patients in the CG, 4 (28.6%) in TG1 and 10 (50%) in TG2 showed an improved outcome. At T2 the following improvement compared to T1 was observed: 8 children in CG, 8 in the TG1 and 9 in TG2. At the end of the three-month observation period 14 patients (70%) in CG, 12 (85.6%) in TG1 and 19 (95%) in TG2 improved the findings of their TMs (Fig. 3).

## Discussion

Overall, our results showed that Adelmidrol as a nasal spray and in combination with a pharyngeal spray can be useful to reduce the inflammation of the URT, especially in case of nasal and ear involvement.

Although traditional treatment efficiently reduced nasal and ear symptoms, Adelmidrol demonstrated a greater improvement, especially when the molecule was administered in combination (oral and nasal) for 3-months.

Traditional therapy (nasal wash with saline solution and nasal aerosol with Fluticasone and Mucolytic) resulted in the same benefit of Adelmidrol, when used as nasal spray alone; on the contrary, the combination of oral and nasal Adelmidrol notably enhanced the outcomes in both nose and ear findings.

We used Adelmidrol in two different formulations: Rinidrol is a nasal spray that contains Adelmidrol and hyaluronic acid; Oridrol is a pharyngeal spray that combines Adelmidrol with extract of Echinacea Angustifolia. Both formulations were in saline isotonic solution.

The additional components of the two products should be considered: hyaluronic acid (in Rinidrol^®^) helps to maintain the hydration of nasal mucosa, improving nasal clearance [12]; Echinacea Angustifolia presents great ability to fight viral infection especially in children [13]. We speculate the combination of an antiviral with Adelmidrol might have improved the efficacy of the spray both at the level of the oropharynx (direct anti-viral effect) and indirectly in the nasopharynx (indirect effect).

Different natural local compounds have been tested for treating URT inflammation and infection; however, to the best of our knowledge, this is the first study that used Adelmidrol in children.

In 2021 a pharyngeal spray with propolis (a natural resinous mixture produced by honeybees**)** was able to reduce the symptoms of uncomplicated URT infection in an adult population [14]. This monocentric, randomized, double-blind, placebo-controlled clinical trial (CT) showed that propolis shortening symptoms remission just after 3 days of use in 83% of case, compared to 72% of subjects in the placebo group. In all cases patients recovered from symptoms by the fifth day. The authors concluded that propolis, thanks to its antimicrobic and anti-inflammatory proprieties, could speed symptoms resolution [14].

A double-blinded CT tested the efficacy of a throat spray containing Teramune Bronchus to promote good health of the respiratory tract in a sample of 35 women (18–60 years) [15]. Women who used the spray presented better outcomes than control [15].

Sixty adults (24 treatment and 35 placebo) were included in a clinical trial using a spray with Eucalyptus citriodora, Eucalyptus globulus, Mentha piperita, Origanum syriacum, and Rosmarinus officinalis [16]; the aim was to evaluate the ability of the product to reduce the symptoms of upper respiratory tract inflammation. No benefits were observed when the multi natural compound spray was compared to the placebo [16].

Looking overall to the results of these studies, pharyngeal sprays with natural compounds seem to be useful in reducing length and severity of the URT infections and inflammation in adults.

Although Oridrol (Adelmidrol in pharyngeal spray) reduced the inflammation in the oropharynx, it did not determine a substantial reduction of the tonsillar tissue to obtain statistically significant results when compared with control. We hypothesize that this may be due to the limited time action (a few seconds) of pharyngeal sprays; in fact, salivary swallowing cleaning the mouth also removes the product from pharynx. This might explain the limited efficacy of Oridrol, but also the poor results obtained by other researchers with similar products [14–16].

Nasal sprays with natural compounds were lesser studied than oral spray. We only found one study evaluating the effects of a nasal spray with natural compounds (*hyllantus* and *Andrographis paniculata)* on URT inflammation, with a particular focus on nasal outcomes. Specifically, the researcher analyzed the effect of this nasal spray, which also contained copper ions and zinc, to fight unspecific viral-induced URT infection [17]. The spray, thanks to its antiviral and immune stimulating proprieties, was able to determine remarkable benefit compared to nasal wash with saline solution [17].

Nasal sprays containing inactive bacteria were successfully tested on children and were able to reduce recurrence of URT infection and inflammation [18]. However, inactive bacteria are not plant extracts, so these studies are not comparable with the present one.

Previous studies analyzed the benefit of natural compounds exclusively on viral/bacterial infections [19]. The molecule we used in the current study, Adelmidrol, acts in a different manner as it modulates the immune response [2, 4, 9] rather than inhibits viral or bacterial replication and diffusion [17, 18]. Moreover, based on the results obtained using PEA, of which Adelmidrol is a precursor molecule, on Sars-COv2 replication inhibition [9], we hypothesized that Adelmidrol could have similar results with common rhinoviruses [9]. The benefit could be also supported by the anti-inflammatory effect of Adelmidrol that improves the nasal environment maximizing the host immune response [9]. In fact, it has been shown that Adelmidrol reduces the airway infiltration by inflammatory cells, Myeloperoxidase (MPO) activity, and pro-inflammatory cytokine overexpression (IL,6 IL-1β, TNF-α, and TGF-1β) [4]. The reduction of inflammation in the adenoids and tonsils [2] improved nasal ventilation and the middle ear ventilation with amelioration of TM findings.

**Fig. 4:**
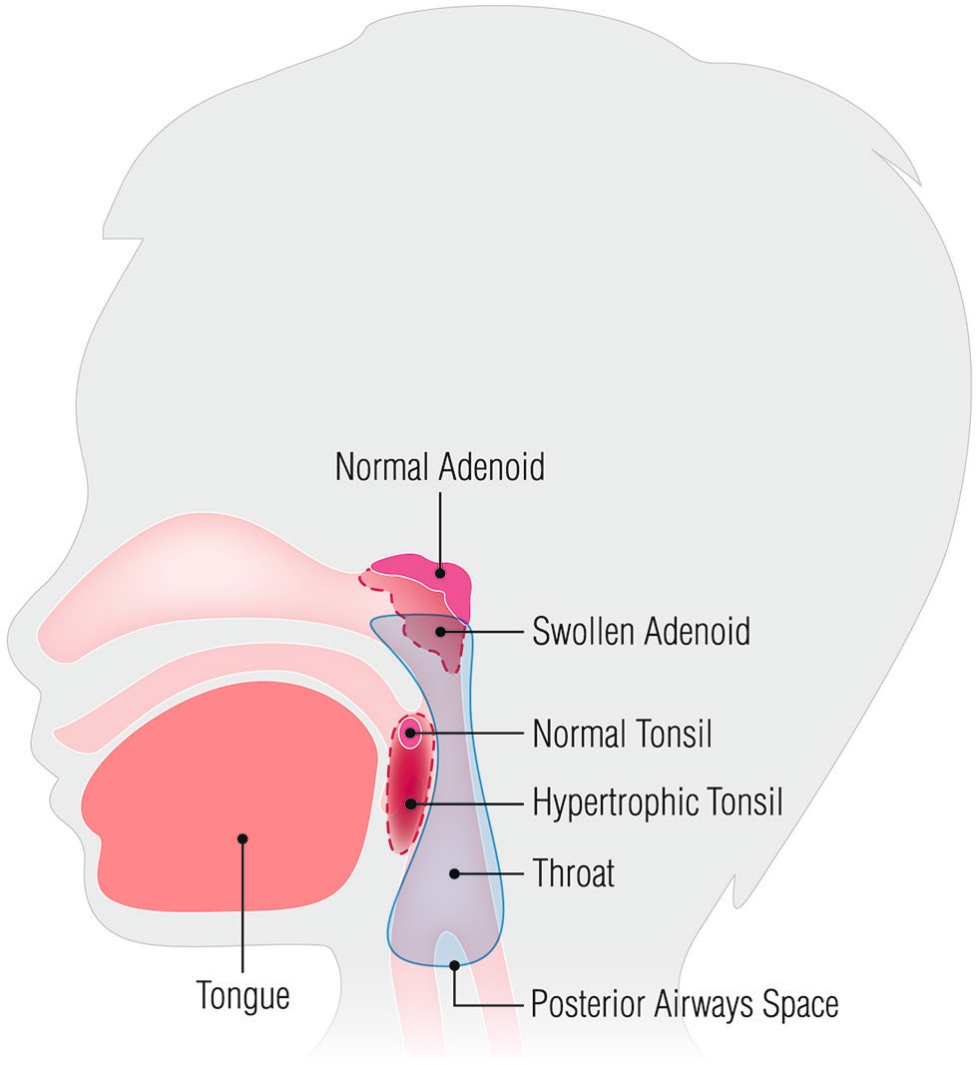
The drawing shows how the hypertrophy of adenoids and tonsils can reduce the posterior airways space and how the hypertrophy of both structures can reduce the ventilation in the middle ear by occluding the exit of Eustachian Tube

The benefits obtained by the combination of oral and nasal Adelmidrol are probably related to the anatomy of the upper airways, in particular the posterior airway space (PAS) and the ET. The former extends from the nasopharynx through the oropharynx to the hypopharynx and has a different diameter from top to bottom [20]. Because the ET exits at the level of nasopharynx, one of the narrow area of PAS [21], the increase in soft tissue dimensions within the oropharynx (such as tonsils) or in the nasopharynx (such as adenoid tissue) can reduce this area, potentially affecting airway function. Moreover, because of the position of the ET, inflammation of adenoids and tonsils can ascend to the middle ear [2]. During mastication and swallowing the contraction of pharyngeal muscles and the opening of the ET allows viruses and excessive secretions, that are present in case of inflammation, to go back up to the middle ear [22]. It has been shown that appropriate treatment of the upper airways can significantly benefit ear health and improve hearing capacity in children affected by otitis media [2, 3]. The reduction of inflammation decreases the presence of secretion on the adenoid tissue, and the adenotonsillar volume. Increased PAS ameliorates middle ear ventilation, and less secretions have less likelihood to go back up to the middle ear (Fig. 4).

This can explain why the combination of oral and nasal Adelmidrol yields better outcomes compared to the CG and nasal Adelmidrol alone.

In our sample Adelmidrol, thanks to its anti-inflammatory proprieties, reduced the adenotonsillar inflammation [7], improved nasal respiration, and freed the PAS, allowing better ventilation of the middle ear and drainage of secretion from the middle ear.

Nasal treatment improved TM findings even in those cases (TG1), in which the patients presented minimal volume reduction of the lymphoid tissues (Fig. 4); the reduction of adenoid hypertrophy prevents the secretion ascending from the nasopharynx into the middle ear, improving TM findings [2, 3]. The exclusive use of Adelmidrol as a nasal spray might have had an indirect anti-inflammatory effect on the tonsils because of the retronasal passage. However, because the product was deposited only on the posterior surface of the tonsil, its benefit was not enough to appreciate improvements. The combination of nasal and oral Adelmidrol spray (TG2) might have directly acted on adenoids and tonsils; we speculate that nasal spray reached the adenoids and the posterior portion of the tonsils, whether the oral spray directly on the anterior pharyngeal portion of the tonsils, thus increasing the Adelmidrol anti-inflammatory effect and efficacy (Fig. 4). This might have speeded the reduction of the inflammation obtaining normal nasal and ear findings after 1 month of treatment only.

It is important to underline that children with adenotonsillitis often have a significant allergic component [21]; it has been observed high concentration of IgE in tonsils and adenoids even in patients with low blood IgE level [21]. Adelmidrol, like PEA, might modulate the local response (particularly into the nose) of mast cells [23], thereby reducing the “allergic component” of the process and facilitating the resolution of URT inflammations [24, 25].

Additional studies that evaluate the local level of inflammation in the nose could be useful to clarify the effect of the nasal spray solution containing Adelmidrol. In fact, only the benefits of PEA on infections and inflammation of the upper airway tract have been widely confirmed [23, 26].

To date, it is only possible to speculate the effect of Adelmidrol in URT inflammations in children. The reduction of the inflammation and the inhibition of the hyperactivation of mast cells might generally explain the positive outcomes observed in the current study.

### Limits of the study

This study has both major and minor limitations.

The first major limitation is that this was a pilot study, performed in a single center study, with small sample size, no placebo group and non-blinded enrollment of the patients. Multicenter studies including larger sample sizes are necessary to confirm these results. The consecutive enrollment of the patients cannot be considered as a real blinded clinical trial, but it must be exclusively considered as control study. Secondly, this was not a double blinded study including patients treated with placebo; in fact, for ethical reason it was not possible to treat children using placebo only. A structured double-blinded clinical trial should be considered for further studies. Furthermore, we only performed otoscopic and fiberoptic endoscopic evaluations, without performing tympanograms or auditory tests. The improvement in otoscopic findings might not correspond to improvements in auditory capacity, although previous studies have shown a direct correlation between changes in the tympanic membrane and auditory tests [2, 3]. Therefore, additional studies that include tympanogram and pure tone audiometry to fully evaluate the auditory function are strongly recommended.

Another important limitation is that otitis media with effusion is quite common and self-limiting in this population, so additional studies that include audiological tests should be performed to understand if the condition has a clinical impact.

Finally, we based our evaluation on Cassano score and we did not perform additional tests,.ie polysomnography, to evaluate the clinical impact of the adenonsillar hypertrophy. Additional studies should be performed adding functional tests to understand the real clinical effect of the therapy.

The first minor limitation was the lack of measurement of changes in the PAS; instead, we only assessed adenoid and tonsil hypertrophy. Any improvement in PAS diameter is purely speculative. Additional studies should objectively measure the volume of PAS before and after treatment. Then, this study was conducted for a limited period (3 months). Longer observational studies are needed to determine if Adelmidrol can significantly reduce the frequency of annual infections.

## Conclusions

This pilot study showed that a combination of oral and nasal Adelmidrol spray could reduce the inflammation of the URT and improve the ear findings in our sample of 60 children. Although the traditional treatment had a similar efficacy to Adelmidrol, it might have adverse events that are avoidable using natural compounds. These results seem promising; however, additional clinical trials should be performed to better evaluate the effect of Adelmidrol on different URT conditions.

## Data Availability

The data are available under reasonable request to the corresponding author.
